# Invasive Insects Differ from Non-Invasive in Their Thermal Requirements

**DOI:** 10.1371/journal.pone.0131072

**Published:** 2015-06-19

**Authors:** Vojtěch Jarošík, Marc Kenis, Alois Honěk, Jiří Skuhrovec, Petr Pyšek

**Affiliations:** 1 Department of Ecology, Charles University in Prague, Prague 2, Czech Republic; 2 Institute of Botany, The Czech Academy of Sciences, Průhonice, Czech Republic; 3 CABI, Delémont, Switzerland; 4 Crop Research Institute, Prague 6-Ruzyně, Czech Republic; United States Department of Agriculture, Beltsville Agricultural Research Center, UNITED STATES

## Abstract

We tested whether two basic thermal requirements for insect development, lower developmental thresholds, i.e. temperatures at which development ceases, and sums of effective temperatures, i.e. numbers of day degrees above the lower developmental thresholds necessary to complete development, differ among insect species that proved to be successful invaders in regions outside their native range and those that did not. Focusing on species traits underlying invasiveness that are related to temperature provides insights into the mechanisms of insect invasions. The screening of thermal requirements thus could improve risk-assessment schemes by incorporating these traits in predictions of potentially invasive insect species. We compared 100 pairs of taxonomically-related species originating from the same continent, one invasive and the other not reported as invasive. Invasive species have higher lower developmental thresholds than those never recorded outside their native ranges. Invasive species also have a lower sum of effective temperatures, though not significantly. However, the differences between invasive and non-invasive species in the two physiological measures were significantly inversely correlated. This result suggests that many species are currently prevented from invading by low temperatures in some parts of the world. Those species that will overcome current climatic constraints in regions outside their native distribution due to climate change could become even more serious future invaders than present-day species, due to their potentially faster development.

## Introduction

Temperature is one of the most important environmental factor affecting insect growth rate, fecundity, mortality and movement [1,2]. Consequently, temperature-based insect phenology models, constructed from the virtually linear relationship between the rate of development and temperature, have long been used to help growers predict pest occurrence or performance of pests’ natural enemies [3,4]. More recently, these models have been used as a component of risk analysis for predicting the establishment and spread of exotic pests [5,6].

Phenology models require knowledge of two thermal requirements, the lower developmental threshold (LDT), i.e. the temperature at which development ceases, and the sum of effective temperatures (SET), i.e. day degrees (D°) above the LDT necessary for completion of a developmental stage. The concept of thermal dependence of development, first suggested nearly three hundred years ago [7], make it possible to predict when a particular stage in the development of an individual insect will be completed. The prediction of phenology model is then based on accumulating D° above LDT, which in most cases is based on daily maximum and minimum temperatures, assuming a sine curve can be used as an approximation of the diurnal temperature curve [8]. By linking these thermal requirements, which define the environmental conditions in which a species can maintain populations, with geo-referenced meteorological data, it is now possible to predict species distributions using deductive climate models [6] such as CLIMEX [9] and NAPPFAST [10].

Insect invasions have been receiving much attention recently both in terms of producing large-scale inventories and identifying underlying mechanisms and impact [11–14]. Recent analyses of large data sets improved understanding of the role of climate in insect invasions. For example, it has been demonstrated that over the second half of the 20th century increasing establishment rates of invasive insects in China were positively related to rising surface air temperature and the relationship remained significant after accounting for increase in international trade [15]. Therefore the increase in establishments of invasive alien insects could be explained only in part by introduction rate and propagule pressure; similar relationships have been also reported from the UK and United States [15]. Warmer temperatures can favor establishment and spread of alien insects by providing new areas suitable for colonization, enabling insects to shift their geographic range polewards, and crossing barriers that previously limited their natural ranges [16–19]. This brings about the need to consider global warming when designing strategies and policies to deal with insect invasions [15]. It seems therefore plausible to focus, at least partly, on species traits related to temperature, to obtain insights into the mechanisms of insect invasions and improve predictions of future invaders.

Although the use of species traits for predicting species invasiveness has been questioned recently [20], available evidence suggests rather the opposite [21–24]. Traits associated with invasion success are commonly used in invasion risk-assessment schemes for alien plants [25], fish [26], mammals [27], birds [28] and specific groups of insects (e.g. [29–33]).

Here we provide an analytical background for using thermal requirements to predict insect invasions, without the need to incorporate these traits into phenological and climatic models. The approach adopted here is based on the assessment of whether the two basic thermal requirements, LDT and SET, differ among related species that are successful invaders and those that are not. For this purpose, we define an invasive species as one that is known to have established self-sustaining populations outside its supposed native range without being intentionally introduced by humans, and a non-invasive as one that is not known to have done so. We evaluated pairs of related species differing in invasiveness because thermal requirements are known to be similar for related taxa [34,35]. Moreover, we use pairs of related species native to the same continent because by focusing on pools of species that originate from the same region, we also partly eliminate other potential biases such as those related to regional variations in the volume of trade and potential for accidental transport between regions. Because the analysed species are mainly pests and their natural enemies, using this so-called source-area approach members of a fauna are deemed relatively comparable in terms of their chance of being transported by humans from their native region to other parts of the world [36,37].

## Materials and Methods

A database of 1,368 primary research studies providing the development times of 659 species of insects and mites at various temperatures was gathered by Alois Honěk and colleagues [38–40]. Lower developmental thresholds (LDTs) and sums of effective temperatures (SETs) were calculated for each insect and each study from the linear relationship between the developmental rate and temperature as described in **S1 File**. This database was merged with a database of thermal requirements gathered by online search of CAB Abstracts between 1972 and 2004 by Nietschke and his colleagues [41], which contain data on LDTs and SETs of more than 500 species of insect, and is being continuously updated. Overall, the merged database of thermal requirements (DTR, [35]) comprised 2,722 original studies of nearly 1,000 species of insects and mites under laboratory conditions, many for several populations, mostly pests and their natural enemies from all over the world (available at https://secure.fera.defra.gov.uk/pratique/publications.cfm?deliverables=1). This DTR was the source of the 100 pairs of species used for comparisons of their thermal requirements (**S2 File**).

Pairs of species originating from the same continent were selected on the basis of their taxonomic relatedness, i.e. the pairs belonged to the same genus, tribe, sub-family or family, in this order of preference. Biological control agents and other intentionally introduced insects were excluded, unless it was clear that their introduction into a new continent was accidental (e.g. several Coccinellidae). Pests of stored products were also excluded because most of them have a worldwide distribution and are cryptogenic, i.e. their area of origin is unclear. Where several independently studied populations of the same species were available, average LDT and SET values from all these populations were used in analyses. When many values were available for the same species, doubtful values that were obviously too different from the others (outliers) were discarded. When several species of similar relatedness were available for comparison, a single non-invasive species was compared with the average value for several related invasive species; we considered this to be more conservative than choosing a single species for comparison subjectively or randomly. For the same reason, non-invasive species were coupled only once but, in a few cases, different non-invasive species were coupled with the same related invasive species (**S2 File**). When possible, for each species pair, SETs for the total (egg to adult) pre-imaginal development were used but, when not available, specific developmental stages were used. Similarly, LDTs based on the whole development were preferred but, when not available, LDTs of any developmental stage (or the average of LDTs of different developmental stages) were taken since all the developmental stages within a population of a species are supposed to have the same LDT [40,42–44]. The sample size for SETs was slightly smaller (n = 88) than for LDTs (n = 100). Most of the LDTs were for species pairs that originated in Europe (n = 36, including species with a Palaearctic distribution) and North America (n = 43, including species with native range extending into Central and exceptionally into the north of South America), and less from Asia (n = 15, mostly originating from East Asia) or other continents (n = 6: Africa, Oceania and South America). The subset of SETs for paired species included 33 European, 35 North American, six Asian and five from elsewhere.

Because the same invasive species was sometimes used more than once for comparison with several native species and because the species relatedness varied within pairs, we used three different statistical analyses of LDTs and SETs to remove potential statistical biases. First, we compared LDTs and SETs of invasive and non-invasive species without taking into account that the differences in LDTs and SETs between pairs can vary specifically depending on their taxonomic affiliation. This analysis, done by two-sample t-test with invasive/non-invasive status of each species as a fixed factor (e.g. [45]), is compromised by the fact that the chosen pairs of invasive and non-invasive species are not independent. On the other hand, it can alleviate the problem of the underestimation of variation of LDTs and SETs for invasive species that were used repeatedly in the same analysis. Second, to reveal taxonomic effects for pairs of closely related invasive and non-invasive species, linear mixed effect models with the taxon to which each pair belongs as a random factor and status of each species (invasive/noninvasive) as a fixed factor were applied. These analyses were analogous to paired t-tests ([46], p. 135–146) but unlike these tests, they also enabled distinguishing, after explaining the part of variation due to differences between the paired species, the part of residual variation within paired species from residual variation between taxa, which can be used to calculate intraclass correlation between non-invasive and invasive species within pairs. Third, we used linear mixed-effect models on nested taxonomic hierarchy that take into account the differences in species relatedness within pairs. In these analyses, the status of each species (invasive/noninvasive) was considered as a fixed factor and insect taxa as a random factor (e.g. [47–49]). To reveal hierarchical effects of taxonomy, likelihood ratio (LR) tests on the nested model structures were performed. The nested models included (i) a model without taxonomy (i.e., with no random effect), (ii) with orders, and (iii) with families within orders. Finally, to investigate the relationship between the two physiological variables, differences in LDT and SET between invasive and non-invasive species were analysed using a Pearson correlation.

Before analyses, normality of the LDTs and SETs was verified graphically, by calculating skewness and kurtosis [45], and by Shapiro-Wilk tests [50]. Fitted models were checked by plotting residuals, including residuals for random effect of taxa, against fitted values. Calculations were done in R 3.1.2, using library lmerTest 2.0–20.

## Results

**Fig 1:**
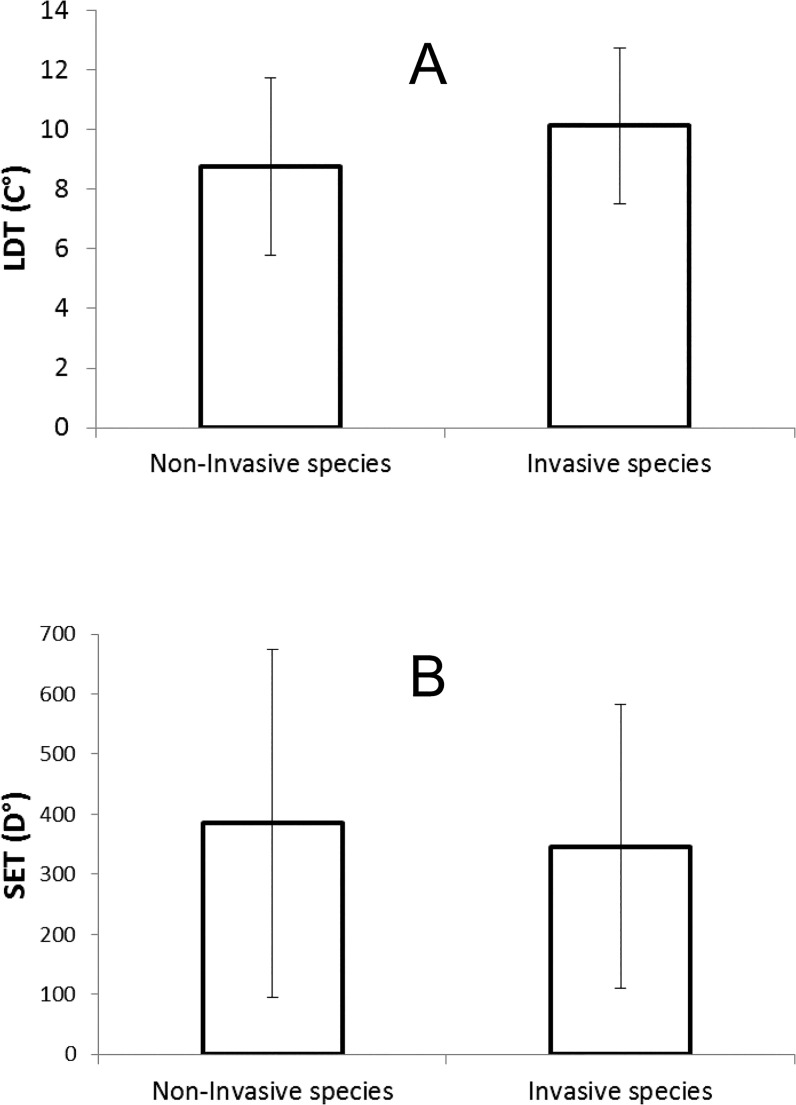
Lower developmental thresholds (LDTs) and sums of effective temperatures (SETs) of non-invasive and invasive species. Average values ± standard deviations of LDTs in °C, (A) and SETs in day degrees [D°] above LDT (B) for pairs of related species of which one is invasive and the other is not. (A) Invasive species have significantly higher LDTs than non-invasive species: t = 4.38, df = 99, P < 0.001 (two-sample t-test not taking into account that paired differences can vary specifically depending on species relatedness); t = 3.841, df = 93, P < 0.001 (linear mixed-effect model on closely related species pairs, analogous to paired t-test); t = 4.35, df = 177.9, P < 0.001 (linear mixed-effect model on nested taxonomic hierarchy). (B) Invasive species have non-significantly lower LDTs than non-invasive species: t = 1.52, df = 87, P = 0.13 (two-sample t-test not taking into account that paired differences can vary specifically depending on species relatedness); t = 1.23, df = 81, P = 0.22 (linear mixed-effect model on closely related species pairs, analogous to paired t-test); t = 1.29, df = 154.9, P = 0.2 (linear mixed-effect model on nested taxonomic hierarchy).

Invasive species had highly significantly higher LDTs than non-invasive species (Fig 1A). The average difference in LDTs of the pairs of species was 1.4°C and invasive species had a higher LDT in 71 out of the 100 pairs. Conversely, the SETs of the species that do not occur outside their native ranges were higher than those recorded for the invasive species in each of the pairs (Fig 1B), though not significantly. The average difference in SET was 39.3 day degrees (D°) and invasive species had a lower SET in 51 out of the 88 pairs (with one pair showing equal SETs).

Although the differences in LDT between the pairs of invasive and non-invasive species were significant even without taking into account that differences within the pairs can vary specifically depending on species relatedness, they became more pronounced when species relatedness was considered (Fig 1). The differences in LDTs were significantly affected both by insect orders (likelihood L = 6.44, df = 1, P < 0.05) and families (L = 12.56, df = 1, P < 0.001), but the intra-class correlation between closely related pairs of invasive and non-invasive species, describing the association between LDTs values within each pair, was relatively low (intra-class correlation ICC = 0.217). The differences in SETs remained non-significant after considering insect orders and families, though models changed significantly by including taxonomic information for orders (L = 15.14, df = 1, P < 0.001). Families nested within orders did not provide additional information (L = 23.12, df = 1, P < 0.0001), and the association between SETs values within each pair was rather weak (ICC = 0.222).

Differences in LDT and SET between invasive and non-invasive species were significantly inversely correlated (R = -0.276; p = 0.009; n = 88), 77% of the pairs having a higher LDT and a lower SET, or vice-versa.

## Discussion

**Fig 2:**
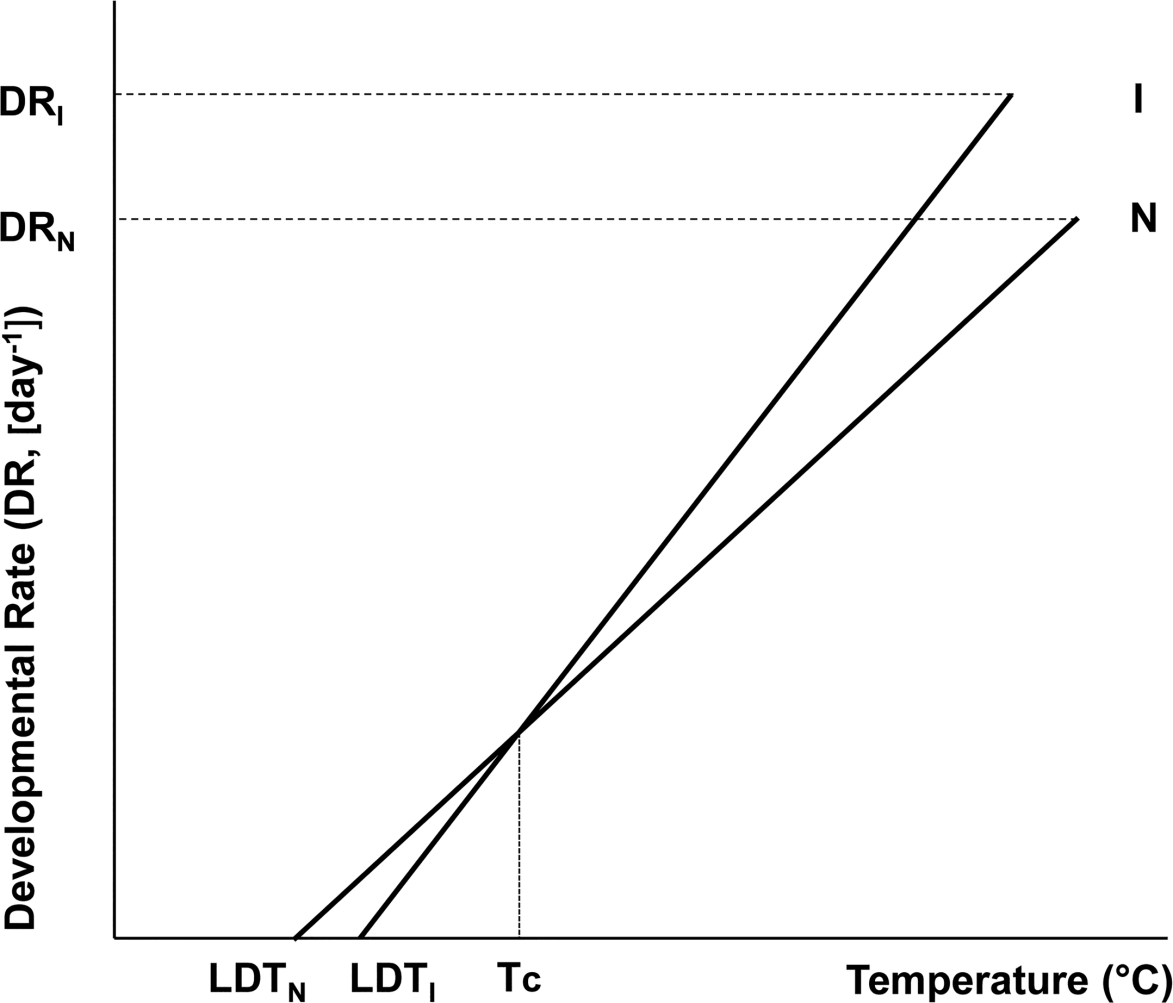
Model of the differences in thermal requirements of invasive (I) and related non-invasive species (N). General model based on the linear relationship between the developmental rate and temperature. Invasive species have a higher lower developmental threshold (LDT_I_) than non-invasive species (LDT_N_), i.e. a higher temperature at which the development ceases. However, as shown by the lines describing the increasing development rate of invasive (DR_I_) and non-invasive (DR_N_) species with increasing temperature, above temperature T_c_ the invasive species develop faster than non-invasive species. Because the sum of effective temperatures (SET) necessary for a completion of a development is a reciprocal value of the slope of the developmental rate on temperature (**S1 File**), faster development means lower SET for invasive than non-invasive species.

It is well recognized that temperature and ongoing global change play a role in insect invasions (e.g. [15–17]). Better information on traits related to temperature could thus improve our understanding of mechanisms underlying the dynamics of the invasion process [51]. Our results indicate a link between invasion potential of insect species and their temperature requirements. In particular, invasive species clearly have a higher LDT than related non-invasive species. The majority of pairs also had a lower SET for the invasive species, but the differences were not significant. There are indications in the literature of a general tendency for the LDTs and SETs of related species to vary inversely [52–53] and this inverse relationship also holds for all the insect data in the original database of thermal requirements used for this study [38,54]. Similarly, in the sub-sample used for this analysis, the differences between invasive and non-invasive species in the two physiological measures were significantly inversely correlated. This suggests that both parameters could act in concert in insect invasions, as described in Fig 2. Invasive species are constrained by their higher LDTs but possibly favoured by their lower SETs, meaning that if the temperature in a region outside their native range is high enough for them to develop they are more likely to become established there than species that are currently non-invasive. The differences in response to temperatures, in terms of LDT and SET acting in concert, indicate that those invasive species that overcome the low-temperature constraints in target regions (they either make it or not, i.e. the qualitative barrier), could well be predisposed to invasion due to their fast development (here the advantage becomes quantitative). However, the relationship between invasive status and SET should be further studied with more pairs, preferably composed of species that are taxonomically closely related (i.e. same genera) and originating from precisely the same area.

Direct use of thermal requirements for evaluating the invasion potential of insects is certainly limited by other factors such as dependences of the survival of the species on their tolerances of cold, wet, dry or heat stress or water availability (e.g. [55–56]). Nevertheless, theory [57] and empirical data [2] indicate that each species can only develop over a limited range of temperatures about 20°C that is relevant for a real life of insects and other ectotherms. In this range, the relationship between the rate of development and temperature is virtually linear (e.g. [58]), and this range is used for calculation of the thermal requirements (**S1 File**). Moreover, the thermal requirements used are those for the LDTs and SETs of non-dormant developmental stages. In particular, the limiting effect of a high LDT may be partly overcome by surviving climatically unsuitable periods in dormant stages [59], which can reduce the limiting effect of low temperatures. If a species enters diapause or hibernates, this has to be incorporated into predictions of invasiveness, which can be further complicated by the fact that, in some species, dormancy can be broken or induced in a new environment [59,60]. Finally, we assume that climate ultimately restricts species distributions. This is a common assumption of phenological and climate-based distribution models because in most situations, climate is the only factor for which data are readily available. However, in reality, the distribution of a species is also under the influence of other environmental components like availability of food, effect of natural enemies, competing species and further habitat-related factors as they are usually included in the concept of the ‘realised niche’ [55,61].

It cannot be ruled out that some of the differences observed in thermal requirement between invasive and non-invasive species may be caused by adaptive evolution in the invaded area, which is commonly observed in biological invasions [62]. In insects, this has been demonstrated, e.g., for the tiger mosquito, *Aedes albipictus* [63], or for parasitoids introduced as biological control agents [64]. Similarly, Preisser et al. (2008) [65] provide evidence for local adaptation to extreme temperatures in the Asian elongate hemlock scale *Fiorinia externa* after its introduction into Eastern North America.

From an analytical point of view, it also needs to be noted that our approach does not point to causality between temperature-related traits of the insect species studied and their invasiveness. Even within the closely related species pairs, other traits correlated with fast development and high LDT could also contribute to separation of invasive species from non-invasive ones. Nevertheless, the differences in LDT between invasive and non-invasive species are more pronounced if species relatedness is explicitly taken into account.

That invading species are limited by their temperature requirements was previously suggested for springtails, based on comparison of egg development times of three native and four invasive species on sub-Antarctic Marion Island; in this particular case, invasive species developed faster but the differences in LDTs were not significant [66]. Temperature barriers to invasion are suggested also for plants, based on both congeneric comparisons and comparisons of whole floras. Alien plants differ in flowering phenology from native species [67–69] and the ability to flower early and/or flower over a long period is a common trait of invasive plants. This indicates that in regions that are climatically colder, many alien species do not become established because they fail to complete their life cycle [21,68]. The role of climate in determining invasions is also demonstrated by the fact that it along with economic factors has shaped the patterns of regional richness of alien birds, mammals and aquatic invertebrates in Europe [70].

Because lower developmental thresholds increase with mean environmental temperature [53–54], this relationship may give insight into the consequences of climate change. Should the temperature in a particular region increase, species that are at present prevented from invading would no longer be limited by low temperatures and a general increase in global temperatures may possibly result in a greater species pool available for invasions globally.

As there are good data for some continents, e.g. [13], predictions of the effects of climate change on invasions by insects could be made for particular regions or species. Although it is not possible to make robust statements, based on the currently available data, about continent-specific patterns, this is a promising venue for future research if more data are specifically collected for understudied regions [71]. Nevertheless, insects that are currently non-invasive clearly differ from related invasive species in important physiological traits that determine their thermal requirements. The fact that thermal requirements that describe the response of insects to temperature, i.e. the environmental factor most affecting their life history traits, differ among related native and invasive species, contradicts views that traits cannot be used to predict invasiveness [20], and provides additional evidence in support of the importance of species origins in biological invasions e.g. [22,24].

## Supporting Information

S1 File Calculation of the lower developmental threshold and the sum of effective temperatures. (DOC)

S2 File Pairs of related species to compare lower developmental thresholds (°C) and sum of effective temperatures (SET in day degrees, D°). (DOC)
