# Supplementary material for: Invasive Insects Differ from Non-Invasive in Their Thermal Requirements
Source: PLoS One. 2015 Jun 19;10(6):e0131072. doi: 10.1371/journal.pone.0131072 (PMC4475049; doi:10.1371/journal.pone.0131072)
Supplement: S1 File — (DOC) [file pone.0131072.s001.doc]

**Supporting information S1:**

**Calculation of the lower developmental threshold and the sum of effective temperatures**

Ectotherm organisms, i.e. all organisms except birds and mammals, rely on external sources of heat for their development. Within a relevant range of temperatures of about 20 °C [1,2], in which the ectotherms actually live, the relationship between the rate of development and temperature is virtually linear [3]. This enables one to calculate two thermal constants: lower developmental threshold, LDT, and sum of effective temperatures [4].

If there is a linear relationship between the developmental rate (DR; i.e., proportion of development occurring per unit time) and temperature, *t*, then

DR = *a +* *bt*, (1)

where *a* is the intercept with the *Y*-axis and *b* is the slope of the linear function. From equation (1), the lower developmental threshold (LDT, i.e., the temperature at which development ceases; RD = 0, *t* = LDT) can be estimated:

LDT = -*a*/*b* (2)

Graphically, LDT is the value at which the relationship intercepts the *X*-axis. Using equation (1), the sum of effective temperatures (SET; i.e. number of day-degrees above the LDT necessary for the completion of a particular developmental stage; DR = 1, *t* = SET, *a* = 0) can also be estimated:

SET = 1/*b* (3)

**References**

1. Charnov EL, Gillooly JF (2003) Thermal time: body size, food quality and the 10 °C rule. Evol Ecol Res 5: 43–51.
2. Dixon AFG, Honěk A, Keil P, Kotela MAA, Šizling AL, Jarošík V (2009) Relationship between the minimum and maximum temperature thresholds for development in insects. Func Ecol 23: 257–264.
3. Sharpe PJH, DeMichele DW (1977) Reaction kinetics of poikilotherm development. J Theor Biol 64: 649–670.

4. Campbell A, Frazer BD, Gilbert N, Gutierrez AP, Mackauer M (1974) Temperature requirements of some aphids and their parasites. J Appl Ecol 11: 431–438.
