# Supplementary material for: Invasive Insects Differ from Non-Invasive in Their Thermal Requirements
Source: PLoS One. 2015 Jun 19;10(6):e0131072. doi: 10.1371/journal.pone.0131072 (PMC4475049; doi:10.1371/journal.pone.0131072)
Supplement: S2 File — (DOC) [file pone.0131072.s002.doc]

**Supporting information S2:**

**Pairs of related species to compare lower developmental thresholds (°C) and sum of effective temperatures (SET in day degrees, D°)**

References and the values for which the averages were calculated are given in a database of thermal requirements described in [1] and available at <https://secure.fera.defra.gov.uk/pratique/publications.cfm?deliverables=1> Methods for pairing the species are provided in the Material and Methods section in the main text.

At the time of submission, three species used in the comparison were not yet included in the online database: *Lymantra monacha* (L.), *Phyllotreta vittula* (Redtenbracher) and *Pyrrhalta viburni* (Paykull). Data on their thermal requirements can be found in [2,3,4]

**References**

1. Jarošík V, Honěk A, Magarey RD, Skuhrovec J (2011) Developmental database for phenology models: related insect and mite species have similar thermal requirements. J Econ Entomol 104: 1870–1876.
2. Karolewski P, Grzebyta J, Oleksyn J, Giertych MJ (2007) Effects of temperature on larval survival rate and duration of development of *Lymantria monacha* (L.) on needles of *Pinus silvestris* (L.) and of L. dispar (L.) on leaves of *Quercus robur* (L.). Polish J Ecol 55: 595-600.
3. Vig K (2004) Biology of *Phyllotreta* (Alticinae), with emphasis on Hungarian and middle european species. In: Jolivet P, Santiago-Blay JA, Schmitz M eds. New developments in the biology of Chrysomelidae. The Hague, the Netherlands: SPB Academic Publishing. 565-576 pp.
4. Weston PA, Diaz MD (2005) Thermal Requirements and Development of Immature Stages of Viburnum Leaf Beetle, *Pyrrhalta viburni* (Paykull) (Coleoptera: Chrysomelidae). Envir Entomol 34: 985-989.

*Species whose thermal requirements are not provided in the online database (see above and references [2-4])
